# Supplementary figures and images for: 3D model retrieval based on interactive attention CNN and multiple features
Source: PeerJ Comput Sci. 2023 Feb 10;9:e1227. doi: 10.7717/peerj-cs.1227 (PMC10280475; doi:10.7717/peerj-cs.1227)

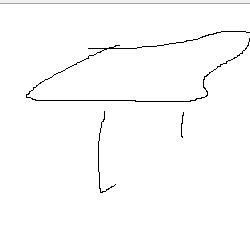

Supplement: Supplemental Information 1 [file peerj-cs-09-1227-s001.zip › sourcecode/D123/picture.jpg]

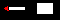

Supplement: Supplemental Information 1 [file peerj-cs-09-1227-s001.zip › sourcecode/D123/res/toolbar1.bmp]

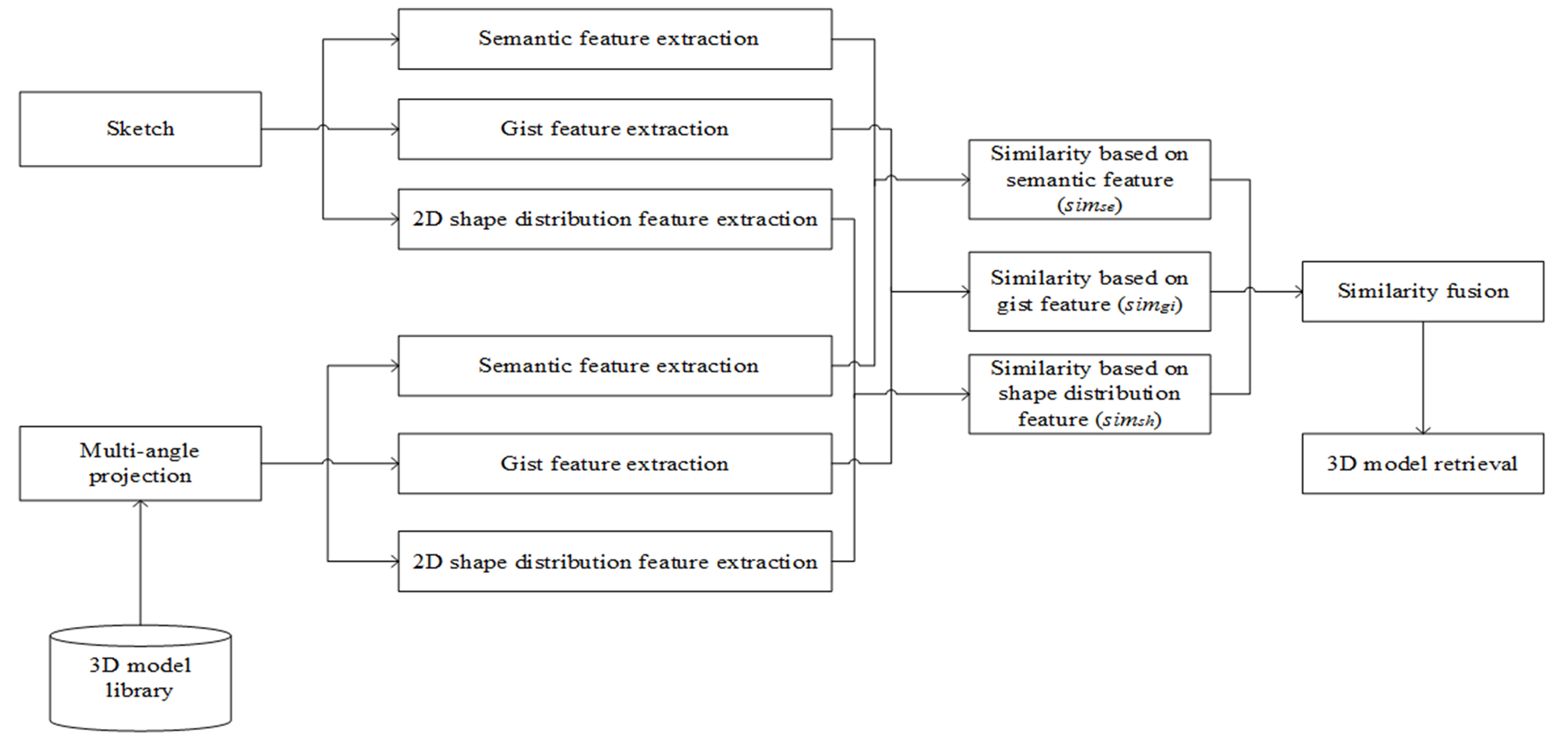

Supplement: Supplemental Information 2 [file peerj-cs-09-1227-s002.png]

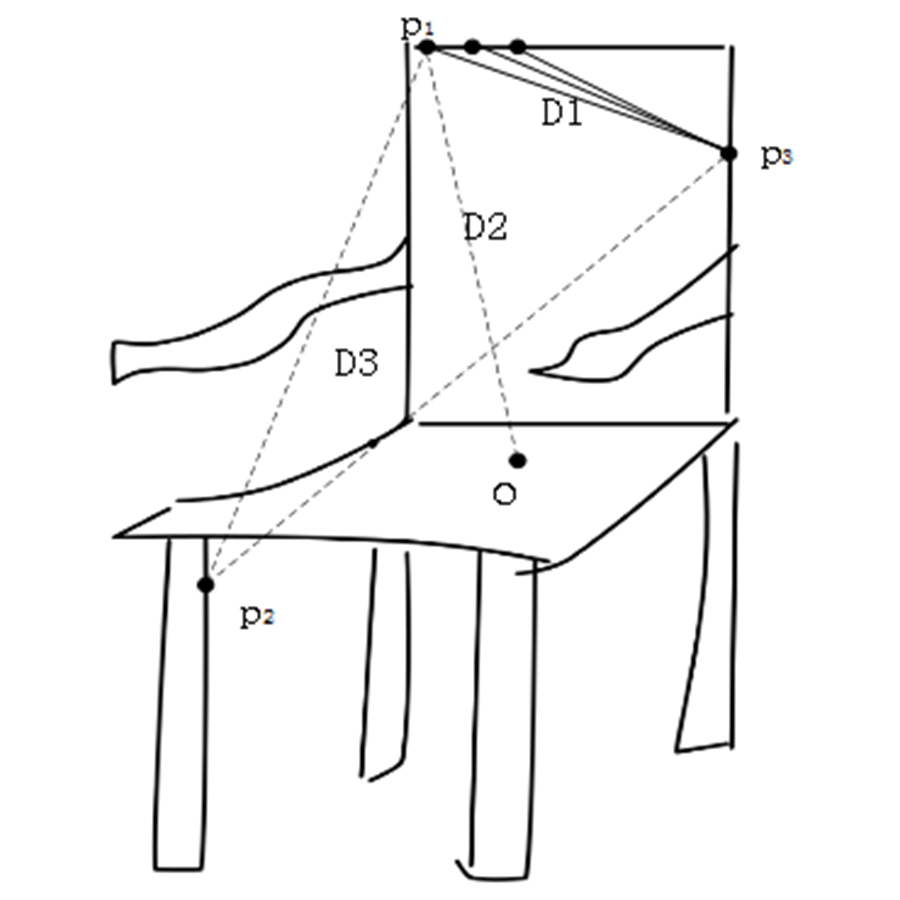

Supplement: Supplemental Information 3 [file peerj-cs-09-1227-s003.png]

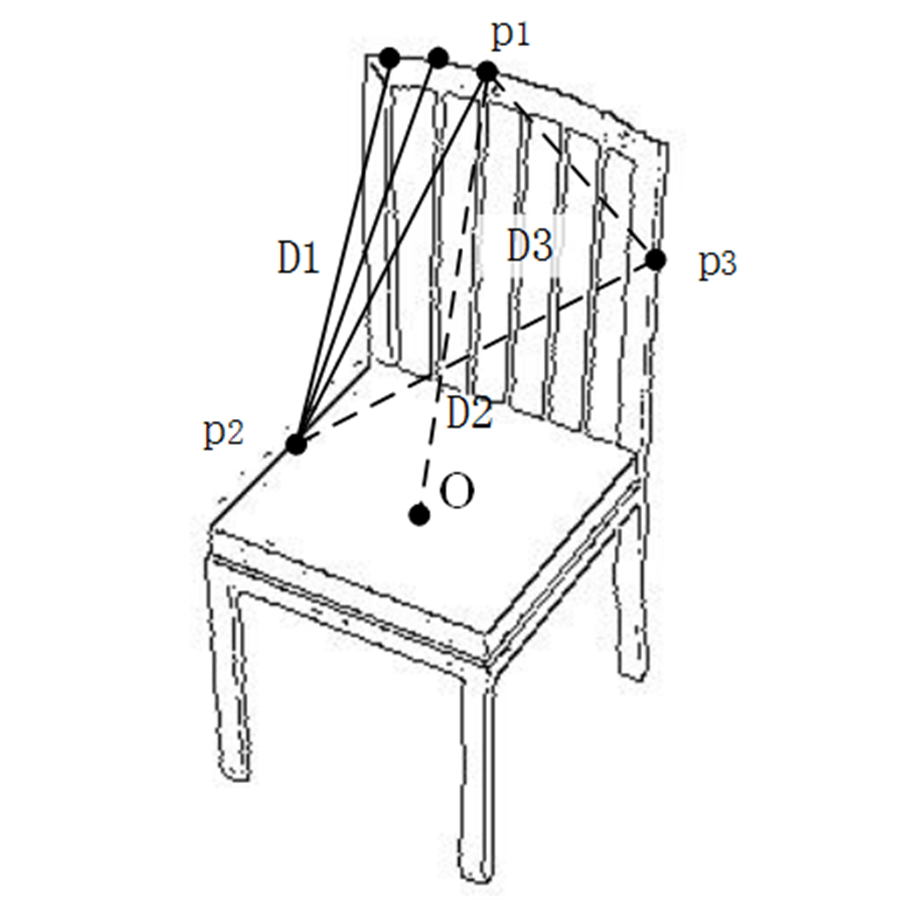

Supplement: Supplemental Information 4 [file peerj-cs-09-1227-s004.png]

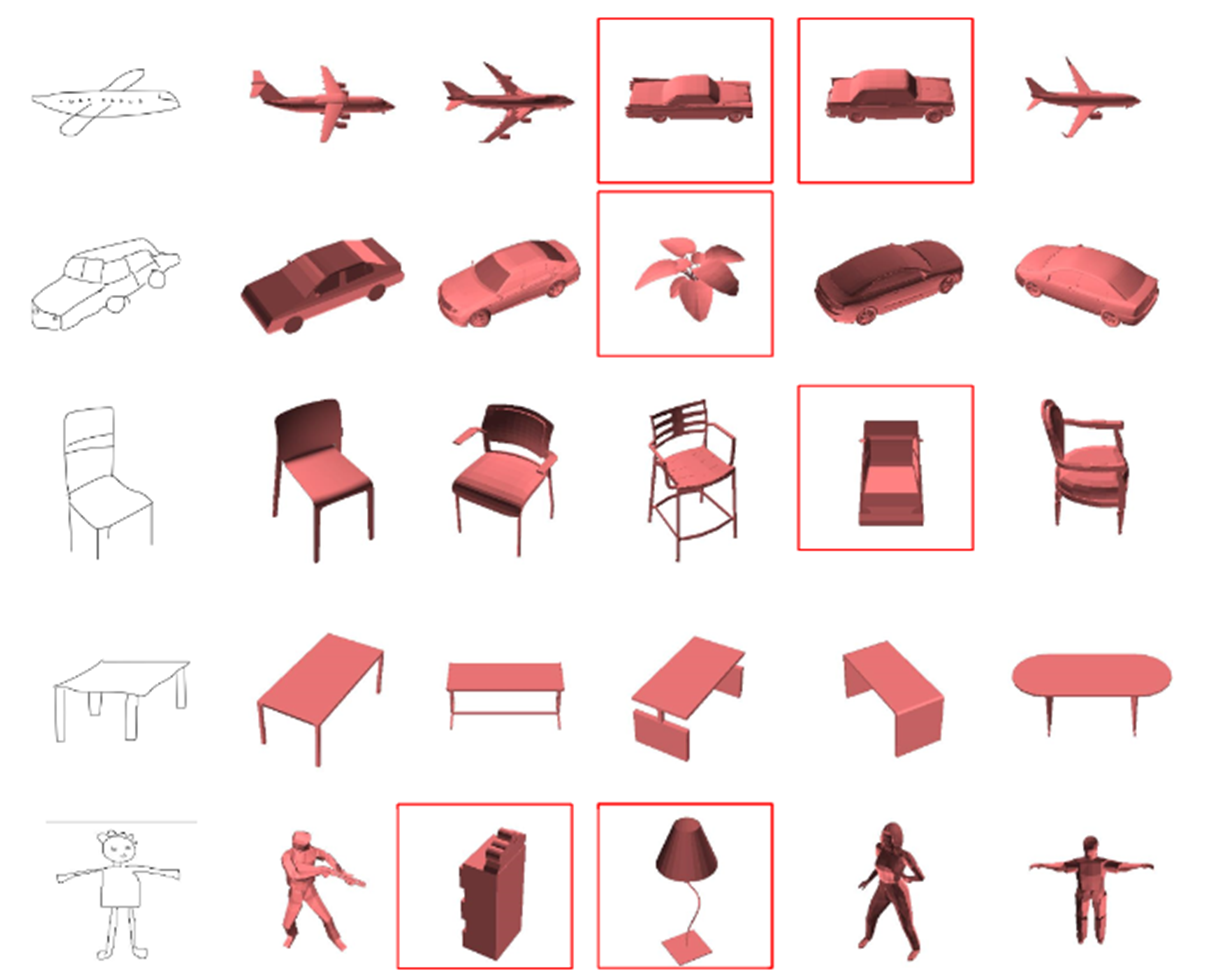

Supplement: Supplemental Information 5 [file peerj-cs-09-1227-s005.png]
