# Supplementary material for: 3D model retrieval based on interactive attention CNN and multiple features
Source: PeerJ Comput Sci. 2023 Feb 10;9:e1227. doi: 10.7717/peerj-cs.1227 (PMC10280475; doi:10.7717/peerj-cs.1227)
Supplement: Supplemental Information 6 [file peerj-cs-09-1227-s006.docx]

| Network layer | Size of convolution kernel | Number of convolution kernels | Size of output |
| --- | --- | --- | --- |
| Conv1 | 11*11 | 32 | 224*224*32 |
| Pooling1 | 2*2 | 1 | 112*112*32 |
| Conv2 | 3*3 | 32 | 112*112*32 |
| Pooling2 | 2*2 | 1 | 56*56*32 |
| Conv3 | 3*3 | 64 | 56*56*64 |
| Concatenate1 | - | - | 56*56*96 |
| Pooling3 | 2*2 | 1 | 28*28*96 |
| Conv4 | 3*3 | 128 | 28*28*128 |
| Conv5 | 3*3 | 128 | 28*28*128 |
| Pooling4 | 2*2 | 1 | 14*14*128 |
| Dropout1 | - | - | 56*56*96 |
| flatten | - | - | 1*1*25088 |
| fc | - | - | 1*1*512 |
| Dropout2 | - | - | 1*1*512 |
