# Supplementary material for: 3D model retrieval based on interactive attention CNN and multiple features
Source: PeerJ Comput Sci. 2023 Feb 10;9:e1227. doi: 10.7717/peerj-cs.1227 (PMC10280475; doi:10.7717/peerj-cs.1227)
Supplement: Supplemental Information 7 [file peerj-cs-09-1227-s007.docx]

| Measurement method | NN | FT | ST | F |
| --- | --- | --- | --- | --- |
| Euclidean distance | 0.889 | 0.544 | 0.756 | 0.469 |
| Chebyshev distance | 0.780 | 0.530 | 0.740 | 0.515 |
| Cosine distance | 0.670 | 0.420 | 0.700 | 0.462 |
| Pearson coefficient | 0.560 | 0.420 | 0.710 | 0.480 |
| braycurtis distance | 0.780 | 0.530 | 0.730 | 0.533 |
